# A phylogenetic method linking nucleotide substitution rates to rates of continuous trait evolution

**DOI:** 10.1371/journal.pcbi.1011995

**Published:** 2024-04-24

**Authors:** Patrick Gemmell, Timothy B. Sackton, Scott V. Edwards, Jun S. Liu

**Affiliations:** 1 Department of Organismic and Evolutionary Biology, Harvard University, Cambridge, Massachusetts, United States of America; 2 Department of Statistics, Harvard University, Cambridge, Massachusetts, United States of America; 3 FAS Informatics Group, Harvard University, Cambridge, Massachusetts, United States of America; University of Chicago, UNITED STATES

## Abstract

Genomes contain conserved non-coding sequences that perform important biological functions, such as gene regulation. We present a phylogenetic method, PhyloAcc-C, that associates nucleotide substitution rates with changes in a continuous trait of interest. The method takes as input a multiple sequence alignment of conserved elements, continuous trait data observed in extant species, and a background phylogeny and substitution process. Gibbs sampling is used to assign rate categories (background, conserved, accelerated) to lineages and explore whether the assigned rate categories are associated with increases or decreases in the rate of trait evolution. We test our method using simulations and then illustrate its application using mammalian body size and lifespan data previously analyzed with respect to protein coding genes. Like other studies, we find processes such as tumor suppression, telomere maintenance, and p53 regulation to be related to changes in longevity and body size. In addition, we also find that skeletal genes, and developmental processes, such as sprouting angiogenesis, are relevant.

## Author summary

Biologists hope to use data from diverse species to identify the genetic basis of continuous traits such as lifespan or beak shape. To do so, they need methodologies that relate genotypic and phenotypic evolution, while taking account of the relationship between species. The practice of integrating data from many species in this systematic way is relatively new, and existing approaches to the problem are often ad hoc, focus on protein coding genes, or involve discretizing continuous measurements. We avoid these limitations and develop a statistical model and software package that can be used to rapidly analyze alignments with respect to a continuous trait. Our method is illustrated by describing 136,859 conserved non-coding elements from 61 mammalian species with respect to the trait ‘long-lived and large-bodied’. We report on the loci highlighted by our model and describe how our results compare to recent studies taking other methodological approaches. We suggest approaches like ours are an important step towards realizing the potential of data collected from across the animal kingdom, whether the aim is to increase our understanding of natural history or to better understand human biology.

## Introduction

In recent years there have been numerous advances in mapping genes underlying phenotypic traits. Many of these advances have built on the successes and refinements of traditional genetic mapping methods yet, as is articulated by Smith et al. [1], such approaches are often limited to the small number of model organisms amenable to crosses or other genetic manipulations. Recently, alternative phylogenetic approaches driven by comparative genomics have emerged as a useful tool for mapping genes in species not amenable to traditional approaches. Several methodologies have been proposed to associate evolution of genes or genomic regions with changes in phenotypic traits including those of [2–7]. These studies use a variety of genomic signatures as evidence of association with phenotypic evolution, including increases in evolutionary rate, loss of function such as pseudogenization, or wholesale deletion of genes or non-coding regions from the genome. Comparative approaches of this kind (hereafter ‘PhyloG2P’) have proved to be surprisingly powerful at identifying associations between genomic and phenotypic variation in the context of convergent evolution of the phenotypic trait.

With the PhyloG2P research programme in mind, this paper aims to make three contributions. First, we highlight the idea of relating phenotypic and genotypic evolution by linking substitution rate multipliers (for nucleotide changes) to variance multipliers (for changes in a continuous trait). Second, we introduce a specific piece of software, PhyloAcc-C, that applies this approach in the context of conserved non-coding elements (CNEs). Third, we illustrate the PhyloAcc-C software using real data, running it with a set of mammalian CNEs and a lifespan related trait as input, thereby providing an opportunity to discuss its output in the context of other recent PhyloG2P-style studies.

The overarching biological motivation for our study is our interest in evolutionary innovation, and here we are particularly concerned with methods attempting to answer the question ‘which CNEs are related to changes in a continuous trait I care about?’ This is an important question because it has been recognized for decades that there are many highly conserved stretches of non-coding DNA that participate in gene regulation across diverse species [8]. Indeed, such sequences are routinely annotated [9] in the UCSC Genome Browser [10] and may then be related to the evolution of phenotypic traits. To give one example, Booker et al. [11] identified conserved sequences that were accelerated specifically in bats, and showed that a subset acted as limb enhancers in transgenic mice. Their conclusion was that some identified enhancers were potentially instrumental to the evolution of bat wings. This conclusion was reached without modelling the co-variation of the rate of enhancer evolution and key measurements from bat wings. However, it is possible that incorporating measurements such as limb length could highlight additional relevant loci that had experienced more subtle evolutionary trajectories than bat specific acceleration.

More generally, as reviewed by Smith et al. [1], there is widespread interest in relating phenotypic and molecular evolution. This interest is evidenced by the substantial effort put into producing a variety of software packages and studies that quantify the relationship between traits and substitution rates. Examples include Forward Genomics [4] and reverse genomics [3], both of which relate sequence similarity (via correlation, generalized least-squares, or heuristics) to traits (with ancestral values inferred using parsimony algorithms). A methodology with a similar goal is that of Treaster et al. [7], which uses tree topology to model the intuitive notion that comparisons between more closely related species should be less confounded by genetic background than comparisons between more distantly related ones. A recent contribution to this diverse collection of approaches is PhyloAcc [12], a Bayesian phylogenetic approach centred on latent conservation states, which is modified here in this paper. A key feature of the above four approaches is that they deal with discrete traits, and in the case of PhyloAcc, do not explicitly model the trait, instead relying on a priori reconstruction, often under the assumption of convergent gain or loss of a character state.

Methods for studying the relationship between continuous traits and molecular evolution are fewer. Coevol [13] models the co-evolution of continuous traits and rates using a multivariate diffusion process, and does not require user supplied branch lengths, although calibrations can be supplied if desired. One imagines that constraining branching times will sometimes be helpful, especially when the sequences being considered are short and highly conserved, and therefore contain few distinguishing differences, as is the case with mammalian CNEs. Two more empirically focused recent studies are that of Yusuf et al. [14], who study the co-evolution of bill shape and both protein coding and non-coding DNA, and that of Kowalczyk et al. [15], who study the lifespan and body size of mammals. The former study used k-means binning to group branches of a tree based on the rate of trait evolution, and then used a likelihood ratio test to compare nucleotide substitution rates under a global clock model versus a local clock model, with one rate per bin. The latter study used the RERConverge method [6], which correlates relative rates of protein evolution and ancestral state reconstructions of a continuous trait, each estimated separately using maximum likelihood.

Here we describe the PhyloAcc-C model, which connects the evolution of continuous traits and non-coding DNA using a statistically integrated approach. We then illustrate the use of our model by applying it to a mammalian trait previously analyzed using RERConverge, but this time considering CNEs rather than protein coding genes, thereby providing analyses that complement the existing literature.

## Methods

The PhyloAcc-C method follows the general Bayesian approach taken by Hu et al. [12] and modifies it so as to model continuous phenotypic change. In this section, we describe the method in enough detail that it may be recreated, and so that one may understand or modify our open source R/C++ implementation.

### Input

The method relies on four inputs: (1) a rooted phylogeny **T** having *L* leaves, *N* = 2*L* − 1 nodes, and *E* = *N* − 1 edges, and that encapsulates the relationships between species from which trait data is drawn; (2) a multiple sequence alignment of homologous CNE sequences from *L* species (rows) at *S* sites (columns) and which makes up the top *L* rows of matrix **X**_*N*×*S*_, which will also model ancestral nucleotides; (3) a vector of continuous trait measurements observed in the corresponding *L* species and which makes up the first *L* elements of vector **y** = (*y*_1_, …, *y*_*N*_), which will also model ancestral trait values; (4) a rate matrix **Q**_4×4_ and stationary distribution **π** that models the background nucleotide substitution process at putatively neutral sites. The alignment may contain gaps which will be treated as missing data. Both the alignment and the nucleotide substitution parameters can be obtained using standard methods as detailed by Hu et al. [12]. In particular, the rate matrix **Q** is often estimated using methods like PhyloP [16] from an alignment of putatively neutral and easily alignable sites, such as fourfold degenerate sites of protein coding loci.

### Model

Each branch is assigned a conservation state *z*_*i*_ that takes on three values: background (*z*_*i*_ = 1), conserved (*z*_*i*_ = 2), or accelerated (*z*_*i*_ = 3). This categorization of branches into three states follows that of Hu et al. [12], which in turn was based on the approach taken by Pollard et al. [16], who apply a conserved and an accelerated state to branches of a tree in the PhyloP software. Conservation states are not assigned freely but follow a Markov process (see e.g. textbook [17]) from root to tips so that the probability of a transition on a branch from parent *i* to child *j* is $\text{Pr}(z_{j}|z_{i})=\Phi_{z_{i},z_{j}}$, the (*z*_*i*_, *z*_*j*_)th element of matrix

$$\begin{array}{c} \Phi =[\begin{array}{ccc} 1-c & c & 0\\ 0 & 1-a & a\\ 0 & b & 1-b \end{array}] \end{array}$$ (1)

with (*a*, *b*, *c*) ∈ (0, 1)^3^.

The structure of this matrix allows CNEs to become conserved and later accelerated. Because a transition from accelerated back to conserved is also possible (i.e., *b* > 0), bursts of acceleration can occur on internal branches. In principle, other matrices can be used to either constrain or relax the transition between rate categories across the tree.

The conservation state of a branch affects both the nucleotide substitution process and the rate at which a trait evolves. Conservation states modulate nucleotide substitution rates via substitution rate multipliers **r** = (*r*_1_ = 1, *r*_2_, *r*_3_) so that the probability of transition from nucleotide *a* to *b* on branch *i* of length *t*_*i*_ is $\text{expm}\{Q\cdot r_{z_{i}}\cdot t_{i}{\}}_{a,b}$. Similarly, conservation states on branches modulate the magnitude of trait changes along branches via variance multipliers **v** = (*σ*^2^, *β*_2_*σ*^2^, *β*_3_*σ*^2^). Under the model, traits evolve according to normally distributed displacements along the branches of **T** such that cumulative displacements are observed in *y*_1_, …, *y*_*L*_. Displacements have mean 0 and a variance that is proportional to both the branch length *t*_*j*_ and the appropriate variance multiplier so that $y_{j}\;|\;z_{j}∼\text{Normal}(y_{i},t_{j}v_{z_{j}})$ and

$$\begin{array}{c} v_{z_{j}}=\{\begin{array}{cc} \sigma^{2} & \text{if}\;z_{j}=1,i.e.,\;\text{background};\\ \beta_{2}\sigma^{2} & \text{if}\;z_{j}=2,i.e.,\;\text{conserved};\\ \beta_{3}\sigma^{2} & \text{if}\;z_{j}=3,i.e.,\;\text{accelerated}. \end{array} \end{array}$$ (2)

### Joint distribution

Letting *pa*(*i*) denote the parent of node *i*, and assuming that *pa*(*j*) = *pa*(*k*) = *i*, we can write the joint distribution of all quantities under our model as:

$$\begin{array}{cc} P(X,y,z,r,v,\Phi ) & =\prod_{s=1}^{S}\prod_{j=1}^{E}P\left(X_{j,s}|r,z,X_{i,s}\right)\prod_{j=1}^{E}P\left(y_{j}|v,z,y_{i}\right)\prod_{j=1}^{E}P\left(z_{j}|\Phi ,z_{i}\right)\\ & \;\cdot [\prod_{s=1}^{S}\pi_{X_{R,s}}]\;P\left(y_{R}\right)\;P\left(z_{R}\right)\;P\left(r\right)\;P\left(v\right)\;P\left(\Phi \right). \end{array}$$ (3)

The last line of the above product indicates that at the root node *R* the prior probability of observing a nucleotide is given by the (input) stationary distribution *π*, whereas the trait and conservation state are specified directly using a prior, as described below.

### Specification of priors

At the root node, we use Pr(*z*_*R*_ = 1) = Pr(*z*_*R*_ = 2) = 0.5. We follow Hu et al. [12] in this choice because we analyze the same CNE sequences, which were originally identified because they appeared widely conserved under a model (phastCons [9]) with two rate categories, conserved and neutral. We use *y*_*R*_ ∼ Normal(0, 1), although users can make their own choices freely. Priors on *a*, *b*, *c* in matrix **Φ** are uniform distributions (i.e., Beta(1,1)). Because we use the same nucleotide data, *r*_2_ ∼ Gamma(5, 0.04) and *r*_3_ ∼ Gamma(10, 0.2) follow the values in Hu et al. [12]. Priors on log *β*_2_ and log *β*_3_ are Normal(0, 1) which is mathematically equivalent to setting a Normal(0, 2) on log(*β*_3_/*β*_2_), the logarithm of their ratio. We also assume that log *σ*^2^ ∼ Normal(0, 2) *a priori*.

### Bayesian inference procedure

Inference is performed using a Markov chain Monte Carlo procedure, which is a combination of collapsed Gibbs sampling with some Metropolis within Gibbs steps [18, 19]. The procedure is a minor modification of that introduced in pages 5–8 of the SI of Hu et al. [12], with the addition of an extra Metropolis step (Step 1), and the introduction of emission probabilities for **y** when sampling **z** (Step 3). The following steps are repeated:

#### Step 1: Sample ancestral trait values y and trait variance multipliers v

Perform Metropolis steps (default is 500) to propose and update *σ*^2^, *β*_2_, *β*_3_, and latent **y**. On 60% of iterations proposals to modify *σ*^2^, *β*_2_, and *β*_3_ are made; on the remaining occasions proposals to perturb latent *y*_*L*+1_, …, *y*_*N*_ are made.

#### Step 2: Sample ancestral nucleotides X

First use the familiar pruning algorithm [20] to calculate the likelihood of subalignment {**X**_*i*,*s*_} rooted at node *i* for all sites *s* = 1…*S* using the recurrence:

$$\begin{array}{cc} P(\left\{X_{i,s}\right\}|X_{i,s},z,r)= & \sum_{X_{j,s}}P\left(X_{i,s}\to X_{j,s}|r_{z_{j}}\right)P\left(\left\{X_{j,s}\right\}|X_{j,s},z,r\right)\\ & \cdot \sum_{X_{k,s}}P\left(X_{i,s}\to X_{k,s}|r_{z_{k}}\right)P\left(\left\{X_{k,s}\right\}|X_{k,s},z,r\right). \end{array}$$ (4)

Next, forward sample ancestral nucleotides **X**_*L*+1…*N*,1…*S*_. For sites at the root node we have *P*(*X*_*R*,*s*_|{**X**_*R*,*s*_}, **z**, **r**) ∝ *P*({**X**_*R*,*s*_}|*X*_*R*,*s*_, **z**, **r**)*P*(*X*_*R*,*s*_) and $P(X_{R,s})=\pi_{X_{R,s}}$ by assumption. For the remaining internal nodes we work from root to tips on a per-site basis using:

$$\begin{array}{c} P\left(X_{j,s}|X_{i,s},z,r\right)\propto P\left(\left\{X_{j,s}\right\}|X_{j,s},z,r\right)P\left(X_{i,s}\to X_{j,s}|r_{z_{j}}\right). \end{array}$$ (5)

#### Step 3: Sample per-branch latent conservation states z

First, from tips to root, calculate the joint likelihood of trait values and nucleotide emissions {**XY**_*i*_} occurring on the subtree rooted at node *i* using the recurrence:

$$\begin{array}{cc} P(\left\{{XY}_{i}\right\}|X_{pa(i),1\ldots S},y_{pa(i)},z_{i},r,v,\Phi ) & =\prod_{s=1}^{S}P\left(X_{pa(i),s}\to X_{i,s}|r_{z_{i}}\right)\\ & \;\cdot P(y_{pa(i)}\to y_{i}|v_{z_{i}})\\ & \;\cdot \sum_{z_{j}}P\left(z_{i}\to z_{j}|\Phi \right)P\left(\left\{{XY}_{j}\right\}|X_{i,1\ldots S},y_{i},z_{j},r,v,\Phi \right)\\ & \;\cdot \sum_{z_{k}}P\left(z_{i}\to z_{k}|\Phi \right)P\left(\left\{{XY}_{k}\right\}|X_{i,1\ldots S},y_{i},z_{k},r,v,\Phi \right). \end{array}$$ (6)

Next, sample **z** from root to tips. At the root our (domain specific) prior is *P*(*z*_*R*_) = (0.5, 0.5, 0.0). For descendant nodes the appropriate probabilities are:

$$\begin{array}{c} P\left(z_{j}|X,y,z_{i},r,v,\Phi \right)\propto P\left(\left\{{XY}_{j}\right\}|X_{i,1\ldots S},y_{i},z_{j},r,v,\Phi \right)P\left(z_{i}\to z_{j}|\Phi \right). \end{array}$$ (7)

#### Step 4: Sample per-category nucleotide substitution rate multipliers r

Perform Metropolis step to propose/update substitution rate multipliers *r*_2_ and *r*_3_.

#### Step 5: Sample latent rate category transition probabilities Φ

The beta prior on entries *a*, *b* and *c* of **Φ** leads to a beta posterior. For example, the posterior of *c* is directly sampled based on **z** transitions from 1 → 2 as follows:

$$\begin{array}{c} c∼\text{Beta}\;(c_{\alpha }+\sum_{j}^{E}I\left(z_{i}=1,z_{j}=2\right),c_{\beta }+\sum_{j}^{E}I\left(z_{i}=1,z_{j}=1\right)) \end{array}$$ (8)

The posteriors of *a* and *b* are calculated similarly using the count of transitions 2 → 3 and 3 → 2 respectively.

### Model selection and ranking of associated loci

A collection of candidate elements can be ranked for association with a trait of interest using the Bayes factor (BF) [21] in favour of the ‘full model’ described above. In the full model, *σ*^2^, *β*_2_, and *β*_3_ are free to vary whereas in the more restricted null model this is not the case, and *β*_2_ = *β*_3_ = 1 so that no systematic relationship between the rate of trait evolution and relative substitution rates is specified. As the null model is nested and the priors on *β*_2_ and *β*_3_ are common to all candidate elements, the BF is estimated using the posterior density of (log *β*_2_, log *β*_3_) at (0, 0). This is an application of the Savage–Dickey method, which is explained in the tutorial of Wagenmakers et al. [22].

### Molecular data

We obtained mammalian CNE alignments directly from the first author of [12], who had in turn originally obtained them from the UCSC 100-way vertebrate alignment [23] available at: http://hgdownload.soe.ucsc.edu/goldenPath/hg38/multiz100way. We obtained the rate matrix and mammal phylogeny (see S1 Text) used to model the background relationship between species from the PhyloAcc GitHub repository, prepared by Hu et al. [12], and available at: https://github.com/phyloacc/Hu-etal-2019-data/. The mammal phylogeny was originally prepared by Murphy et al. [24].

### Software implementation

The PhyloAcc-C software is implemented as an R package [25] that makes use of C++ functions [26, 27] to perform MCMC sampling. To use the package one must load an alignment (e.g. a FASTA file) and a tree (e.g. a New Hampshire file) by using the package ape [28] or similar. Trait data should be loaded into an R data frame and labelled so that it can be matched to the species names in the tree.

The PhyloAcc-C package includes helper functions (sim_X, sim_y, and sim_z) enabling one to simulate alignments, traits, and conservation states under the PhyloAcc-C model. We used these functions to assess model performance in this paper, and a user may do the same given their own phylogeny and rate matrix.

The software, installation instructions, and a tutorial covering simulation and inference are all available at https://github.com/phyloacc/PhyloAcc-C.

## Results

To demonstrate that it is possible in principle to relate the rate of trait evolution to the rate of nucleotide evolution using PhyloAcc-C, we performed a simulation study under ideal circumstances. Then, to illustrate the method using real data, we downloaded principal component data representing the trait ‘long-lived large-bodied’ (LLL) previously studied with respect to protein evolution by Kowalczyk et al. [15]. This trait was analyzed in relation to CNEs previously studied by Hu et al. [12].

In both simulations and our illustrative example, we focused on the quantity log(*β*_3_/*β*_2_). This quantity contrasts variation of a trait on branches undergoing accelerated sequence evolution against variation on branches undergoing conserved sequence evolution; a positive quantity associates faster nucleotide evolution with faster trait change whereas a negative quantity associates faster nucleotide evolution with slower trait evolution. Values close to zero suggest no strong systematic relationship under the PhyloAcc-C model.

Note that the state of a trait on roughly half of all nodes of a tree is unobserved, so it is difficult to make inferences about *β*_2_ or *β*_3_ separately. This is because, in general, a path through a tree can involve sequential switching between periods of hidden evolution under either the *β*_2_ or the *β*_3_ regime. For this reason, we focus on the ratio of the two parameters, for which we can do inference. The logarithm of the ratio is reported because we are interested in the multiplicative effects of the parameter pair, e.g., variance multiplier pair (0.2/0.4) should be thought of in the same way as pair (0.6/1.2) as in both cases the second multiplier is double the first.

### Simulations on full binary trees

Using a fully bifurcating ultrametric tree with 128 tips, we simulated 100 times from our prior distribution, generating latent conservation states, and corresponding DNA sequence alignments and phenotypic trait values. The length of the simulated elements was 80 bp, reflecting the median length of mammalian CNEs in our data set. Branch lengths were all set at 0.1, so that root to tip distances were similar to the longer of the root to tip distances on our mammalian tree.

**Fig 1:**
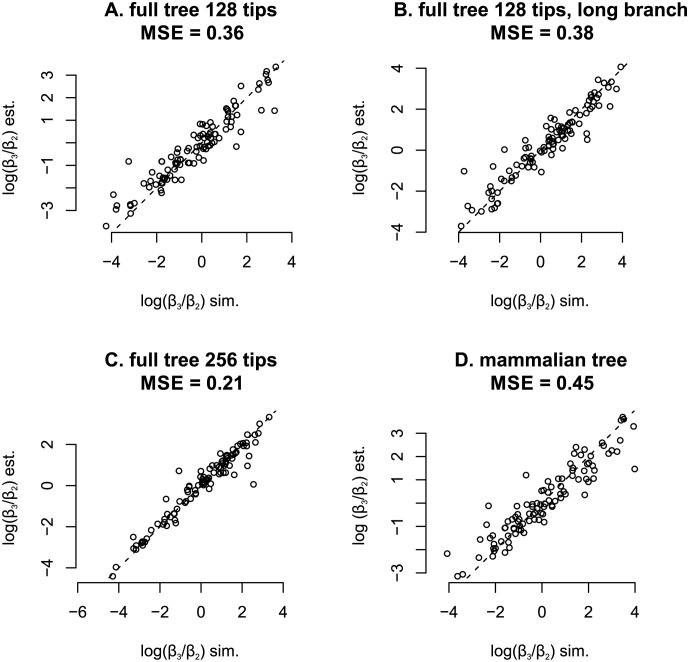
Simulated versus recovered (median) log(*β*_3_/*β*_2_) under model using 80 bp alignments. *A*. fully bifurcating ultrametric tree with 128 tips and all branch lengths set to 0.1; *B*. branch lengths are doubled to 0.2; *C*. tip count is doubled with respect to *A*, but branch lengths are reduced to 0.09 to keep root to tip distance similar; *D*. branch lengths and topology as per mammalian tree (see S1 Text).

We were able to recover log(*β*_3_/*β*_2_) reasonably well, with an MSE (mean squared error) of 0.36 (Fig 1A). The estimates appeared reasonably calibrated: the 80% credible interval (the 10^th^ to 90^th^ percentiles) covered the simulated parameters 72% of the time. FPRs (false positive rates) were characterized by fixing *β*_2_ = *β*_3_ = 1 (no link between trait variation and molecular evolution) and simulating 200 times under two scenarios. In the first scenario (Table 1, col. 2) elements were generated under an exclusively neutral process, in the second scenario (Table 1, col. 5) completely conserved elements were generated. In the latter conserved scenario *r* = 0.2 was used, the expected value of *r*_2_ under our prior. The FPR dropped below 1% in the neutral scenario when a BF of 2 or more was used as a cut-off; under the conserved scenario the corresponding cut-off was also BF ≥ 2.

**Table 1:** FPR when choosing the full model using the BF in favour as a cut-off.

BF cut-off	neutral 128 tips	neutral 128 tips long branch	neutral 256 tips	conserved 128 tips	conserved 128 tips long branch	conserved 256 tips
1	100.0%	99.5%	100.0%	100.0%	97.5%	98.5%
2	0.0%	0.0%	0.5%	0.0%	0.0%	1.0%
3	0.0%	0.0%	0.0%	0.0%	0.0%	0.0%

FPRs were estimated using 200 simulations for each of six scenarios. The tree variants were: 128 tips, branch length 0.1; 128 tips, branch length 0.2; 256 tips, branch length 0.09. The alignment variants used rate multiplier 1 (neutral) and 0.2 (conserved).

We doubled the branch lengths on the bifurcating tree described above to 0.2 and then performed simulations analogous to those described above. We found we were no better able to recover log(*β*_3_/*β*_2_), now seeing an MSE of 0.38 (Fig 1B). The model remained reasonably calibrated as the 80% credible interval (the 10th to 90th percentiles) covered the simulated parameters 74% of the time. FPRs dropped to less than 1% by using BF cut-offs ≥2 in both scenarios (Table 1, cols. 3 and 6).

In the last of our idealized scenarios we doubled the number of tips on the tree to 256 while reducing the branch lengths to 0.09. In this scenario, the recovery of log(*β*_3_/*β*_2_) improved, having an MSE of 0.21 (Fig 1C). The 80% credible interval covered the simulated parameters 85% of the time and BF cut-offs of 3 or more were sufficient to reduce the FPR to less than 1% (Table 1, cols. 4 and 7).

### Simulations on a mammalian tree

Ultimately only performance on real phylogenies with real traits matters. In a manner similar to the above simulations, we took our mammalian tree, having 61 tips, a variety of branch lengths, and an unbalanced topology, and simulated from our prior distributions as before. Recovery of log(*β*_3_/*β*_2_) worked less well, with an MSE of 0.45 (Fig 1D), though the model characterized its uncertainty appropriately as the 80% credible interval (10th to 90th percentiles) covered the simulated parameters 79% of the time.

When testing FPR, we considered scenarios relevant to our size and lifespan results (below). Therefore, we fixed the values of the trait to the real LLL trait values of Kowalczyk et al. [15] i.e. we simulated only conservation states and alignments. Three scenarios were devised: one in which all elements evolved neutrally, one in which elements were conserved at the expected level of *r* = 0.2, and one in which the elements were conserved at *r* = 0.5, which put them above the 99th percentile according to our prior. When simulating short elements (50 bp) against the LLL trait we needed BF cut-offs of 7 (neutral), 3 (conserved), and 8 (barely conserved) in order to reduce the FPR below 1% (Table 2, cols. 2–4); when considering longer elements (180 bp) the relevant BF thresholds were 4, 4, and 7 (Table 2, cols. 4–7). We remark that the barely conserved scenario (*r* = 0.5) presents a challenging set of parameters for the model, which does not mix well when *r*_2_ and *r*_3_ are conflated.

**Table 2:** FPR on the mammalian tree (see S1 Text) when choosing the full model using the BF in favour as a cut-off.

BF cut-off	neutral 50 bp	conserved 50 bp	barely conserved 50 bp	neutral 180 bp	conserved 180 bp	barely conserved 180 bp
1	100.0%	100.0%	100.0%	100.0%	100.0%	100.0%
2	19.5%	1.5%	65.0%	1.5%	2.5%	37.5%
3	2.0%	0.0%	10.5%	0.5%	1.5%	5.5%
4	1.5%	0.0%	2.5%	0.0%	0.0%	2.0%
5	1.0%	0.0%	0.5%	0.0%	0.0%	1.0%
6	0.5%	0.0%	0.5%	0.0%	0.0%	0.5%
7	0.0%	0.0%	0.5%	0.0%	0.0%	0.0%
8	0.0%	0.0%	0.0%	0.0%	0.0%	0.0%

**Fig 2:**
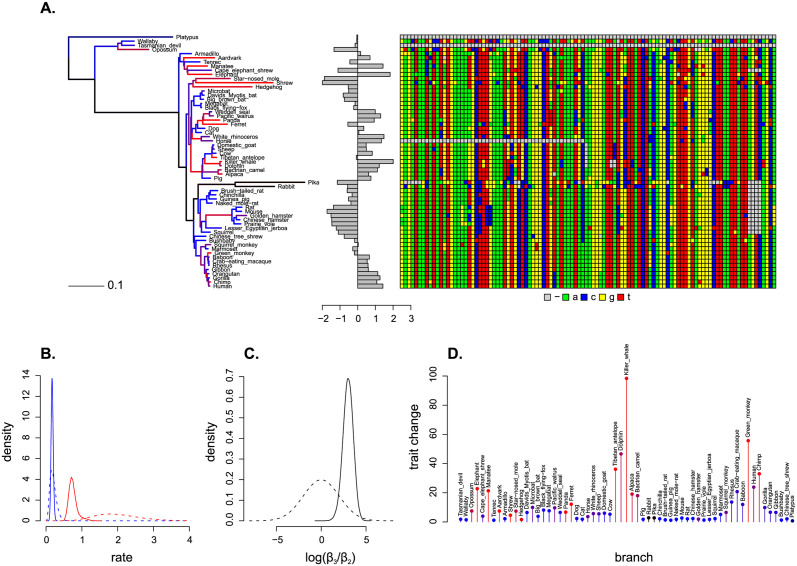
PhyloAcc-C fit to the LLL loci with the highest BF in favour of the full model (VCE277691). *A*. the mammalian phylogeny (input data, see S1 Text) is scaled according to the posterior distribution of rate multipliers **r** and coloured by the posterior distribution of conservation state **z** (black = neutral, blue = conserved, red = accelerated). Next to the tree the LLL trait and CNE alignment (both are also input data) are shown. The corresponding posterior distribution of the trait (i.e. an ancestral reconstruction) is shown in Fig A in S1 Text. *B*. the prior (dashed) and posterior (solid) distribution of the rate multipliers *r*_2_ (blue, conserved) and *r*_3_ (red, accelerated). *C*. the prior (dashed) and posterior (solid) distribution of log(*β*_3_/*β*_2_). In this case the posterior distribution suggests a positive value so that faster nucleotide evolution is associated with faster trait evolution, but see S1 Text for VCE351367 where the opposite is true. *D*. posterior distribution of trait change from tip to immediate ancestor, normalized by branch length and coloured by posterior conservation state. Again note that an accelerated conservation state (red) is associated with bigger trait moves and a conserved conservation state (blue) is associated with smaller ones.

FPRs were estimated using 200 simulations for each of six scenarios. The rate multiplier variants used to generate the alignments were: 1 (neutral); 0.2 (conserved); 0.5 (barely conserved). The alignment length variants were 50 bp and 180 bp. The trait was not simulated but fixed to the LLL values (see Fig 2 or S1 Data).

### Size and lifespan of mammals

Rather than pre-filtering CNE alignments based on heuristics, we instead ran PhyloAcc-C on all alignments with the LLL trait as input and then considered those where the model fit well in a reasonable 10,000 iterations, as assessed via a Gelman and Rubin [29] convergence diagnostic of < 1.01 across 3 chains. This resulted in summaries for 136,859 elements. We ranked the elements by the BF (Bayes factor) in favour of the full model, where the rate of trait evolution is allowed to co-vary with the rate of molecular evolution, versus the null model, where the rate of trait evolution is constant across the phylogeny. We found 30 elements (0.02% of total) where the full model was ‘overwhelmingly’ supported (BF ≥ 100) with respect to the LLL trait and 1,109 (0.81% of total) where the full model was ‘very strongly’ supported (BF ≥30). We note that a BF ≥ 30 generally corresponded to effect sizes of magnitude 2 or more on the log scale i.e. to a ratio of about 7× or more.

The result of running PhyloAcc-C on the element with the highest BF is shown in Fig 2. The ancestral reconstruction of the LLL trait with respect to this element is shown in Fig A in S1 Text. To determine if there were biologically interesting patterns that could be systematically detected based on CNE location, we submitted the 1,109 loci of interest as genomic foreground to a GREAT analysis [30]; the GREAT tool annotates regions of non-coding DNA with biologically meaningful terms using nearby genes, but includes statistical corrections that make it a more principled alternative to an analysis based solely on the gene closest to a given CNE. The full set of 136,859 CNEs (not the whole genome) were used as genomic background for the analysis.

The GREAT analysis suggested no genes were associated with the 1,109 LLL loci of interest, although several GO (gene ontology) biological processes were. These can be summarized as: blood vessel endothelial cell proliferation involved in sprouting angiogenesis; positive regulation of branching involved in lung morphogenesis; regulation of muscle tissue development, differentiation, and proliferation, esp. in the heart; regulation of alkaline phosphatase activity; astrocyte development; organ induction; endocrine pancreas development; trachea formation.

In addition to performing an analysis using GREAT, we also examined the 1 Mbp regions surrounding the top 25 loci associated (via PhyloAcc-C) with the LLL trait using the UCSC [10] and ENSEMBL [31] genome browsers, noting known functions or other annotations of nearby genes. Unlike the GREAT analysis, this was not a statistical analysis, but a set of observations made using the GeneCards tool [32], and linked databases, such as the GWAS Catalog [33]. We found that 12 loci were near genes associated with height, weight, or limb length in some way, mainly via GWAS. Seven loci were associated with cancer genes, seven with the brain or nervous system, six with the skeleton, four with sperm, and one with longevity. Three regions had little to no annotation available whereas four loci were associated with p53, cell fate, or telomere length.

**Fig 3:**
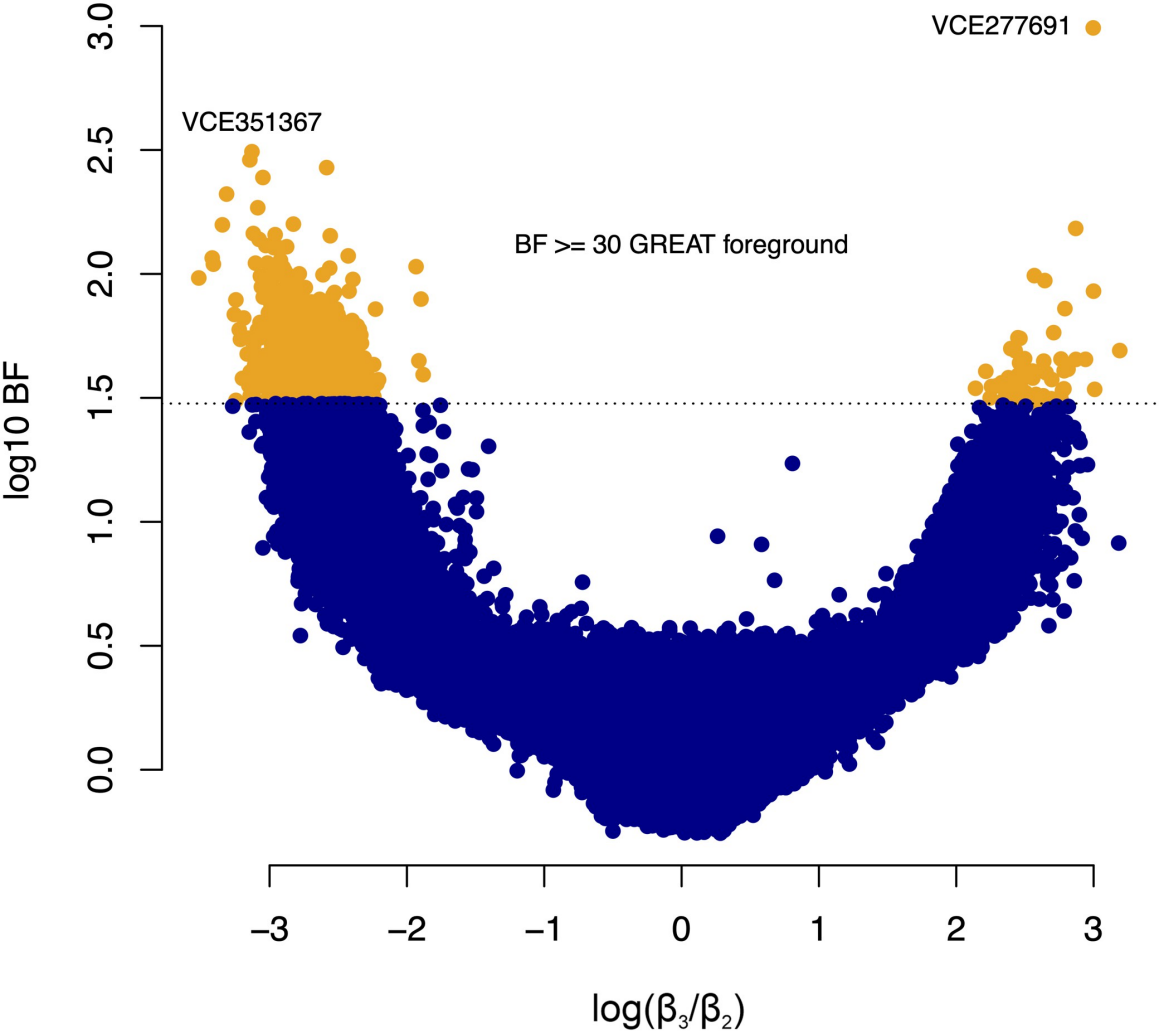
BF versus estimated (median) log(*β*_3_/*β*_2_) for 136,859 mammalian LLL loci. Orange loci are those having BF ≥ 30 and that were submitted as GREAT foreground during analysis. The two loci with the highest BF in favour of the full model are labelled. Note VCE277691 (see Fig 2) and VCE351367 (see S1 Text) have effects with opposite signs.

Overall, LLL loci with BF ≥ 30 exhibited effects in both directions, with log(*β*_3_/*β*_2_) being both positive and negative (Fig 3). The results of our note taking approach, and summaries of the PhyloAcc-C runs on the 136,859 LLL loci are recorded in S1 Data. Full output of the GREAT analysis is reported in S2 Data.

## Discussion

We present a statistical method, PhyloAcc-C, for relating the rate of nucleotide evolution to the rate of evolution of a continuous trait. The model is phylogenetically framed and operates under the common assumption that nucleotide evolution follows a site-independent, continuous-time discrete-state Markov process, and that continuous traits evolve under Brownian motion, although in our case with potentially different rates on different parts of the tree. Latent rate categories are also assigned to each branch using a Markov process, which all together allows the rates of molecular and phenotypic evolution to vary in an automatic way across branches.

A notable feature of the model is its ability to associate the evolution of continuous traits and non-coding DNA using a more statistically integrated approach than that taken by Yusuf et al. [14] or Kowalczyk et al. [15]. Indeed, the general idea of linking genotypic rate multipliers (i.e. evolution relative to a known background tree) and phenotypic rate multipliers (i.e. variance parameters) seems natural, and could be used in other frameworks. For example, the PhyloAcc family of models (https://phyloacc.github.io/) allocates rates with efficient processing of CNEs in mind, yet genotypic and phenotypic rates of evolution can also be linked under more complex models, such as relaxed clocks [34, 35] or local clocks [36], via linear or logistic functions. The efficiency versus accuracy tradeoffs of different rate assignment strategies will not be clear without further research, e.g., allowing substitution rates to change within a branch might be more computationally demanding, but is biologically more realistic and might lead to better model fit, especially because PhyloAcc-C does not take any account of branch length when considering the probability of transition between rate categories.

PhyloAcc-C focuses on linking the rate of genotypic evolution with the rate of phenotypic evolution. This is distinct from: (i) relating the state of a sequence to the state of a trait; (ii) relating rapid sequence change to the state of a trait; (iii) relating the state of a sequence to rapid trait change. Many methods perform analyses related to approach (i), and have been well summarized [1]. Approach (ii) is the domain of Coevol [13] (where fast evolution across 410 mammalian cytochrome b sequences was associated with lower mass and longevity), as well as some more heuristic approaches [14, 15]. In some sense approach (iii) is taken by e.g. reverse genomics [3] and PhyloAcc [12], which both treat sequences in an alignment that are sufficiently different (a threshold) as lost. If one squints hard enough, the coincident loss of a trait can then be considered a rapid change in a trait. However, the aforementioned approaches do not actually model the rate of change of a trait on the lineages where it is lost, so approach (iii) is certainly a potential area for future work.

Simulations show that the model can perform acceptably on both ideal trees and a mammalian tree related to a large set of CNE alignments. This is encouraging, but we suggest users of the method check model performance using their tree, and the sequence lengths and rate multipliers they expect to see. The R software package and instructions accompanying this paper make the simulation process relatively straightforward. In addition, we emphasize that whereas larger trees might provide more data points, there is a judgment call to be made over how large a tree can be plausibly described by three rate categories.

As an illustrative example, we applied PhyloAcc-C to CNEs and longevity data from mammals. From a biomedical perspective, longevity is an important trait, with a long history of study in a diversity of organisms, from worms [37] to humans [38], and therefore there is at least some possibility of assessing the plausibility of candidate loci using published evidence and annotations. Furthermore, lifespan and body-size are also relevant to longstanding conundrums and current ecological debates, including theories of life-history tradeoffs [39, 40], and Peto’s paradox [41], which asks how large and long-lived animals mitigate cancer risk in the face of the many cell divisions that occur during their lifetime. This means the trait is also familiar and of interest to a broad readership. For these reasons, we describe three recent papers studying lifespan that also use broad genomic data, and help put our analysis and methodology into context.

Kowalczyk et al. [15] studied the LLL trait (as previously mentioned, we reuse their trait data) but in the context of protein evolution, with an explicit focus on genes that were interpreted as being under increased purifying selection in long-lived large-bodied species, where LLL was treated as the derived state. Kowalczyk et al. [15] highlighted processes related to the cell cycle, DNA repair, cell death, immunity, and IGF1 expression pathways. Each of these processes were then plausibly linked to lifespan via their analysis. The authors also discuss telomere maintenance and p53, also plausibly linked to aging and cancer control. There is notable overlap between our LLL results and those of [15]: both analyses found associations to p53, telomere maintenance, and cell fate within 1 Mbp of our top 25 loci of interest. Our top 25 loci also have links to cancer and height or body size, though these prevalent diseases and biomarkers are of course heavily studied and consequently commonly annotated, and so we cannot know whether their appearance is simply due to their frequency.

Tejada-Martinez et al. [42] also focused on protein evolution in their study of lifespan and body mass in primates, although they then linked their findings to enhancer evolution. They performed phylogenetic regressions, relating dN/dS to maximum lifespan and body mass for around 10,000 genes. In contrast to Kowalczyk et al. [15], Tejada-Martinez et al. [42] focus on positive (directional) selection on protein-coding genes rather than conservation. The authors identified 276 candidate genes whose rate of adaptive evolution positively correlated with maximum life span in a phylogenetic context. The authors focused their discussion on the enrichment of diverse processes including immunity, inflammation, cellular aging, organismal development (height, BMI), neurodevelopment, and brain function. These processes are all represented in our results in one form or another. None of the genes mentioned in the body of their manuscript occur in our notes on our top 25 loci of interest except for p53, the well known tumour suppressor, which is downregulated by HDAC3, a gene close to the CNE ranked as most-interesting overall in our LLL analysis (Fig 2).

A third study by Treaster et al. [43] takes yet a different approach to understanding longevity. By focusing on 23 species of rockfish that are both closely related and feature a wide range of lifespans (11 to more than 200 years), the authors aimed to identify longevity related protein coding genes while minimizing false positives due to (other) convergent evolution. A key part of the analysis pipeline was the detection of rate shifts using the TRACCER tool [7]. Unlike Kolora et al. [44], and the other studies we mention, Treaster et al. [43] treated longevity as a binary trait and argued against the need to correct for body size. The authors found the ancestral rockfish state to be long-lived, and linked positive selection to glycogen biosynthesis and flavonoid metabolism via GO analysis. The top genes identified in their study do not feature in our notes on our top 25 LLL loci though, as all of us do, the authors find a relationship between their loci of interest and p53, in this case via PLA2R1. We note Treaster et al. [43] and Kowalczyk et al. [15] emphasize insulin signalling pathways, though apparently the particular pathways are under increased constraint in mammals (gene IGF1) but accelerated in Rockfish (gene INSR).

The above studies either focus exclusively on protein coding genes [15, 43] or examine non-coding sequences only insofar as they are identified as byproduct of an analysis with genes as the starting point and main consideration [42]. One distinguishing factor of PhyloAcc-C when compared to these approaches is that its focus is on identifying relevant non-coding sequences. It is possible then that PhyloAcc-C will sometimes identify processes that would otherwise be missed. Indeed, Treaster et al. [43] specifically mention that an attempt was made to analyze the CNE data captured as part of their study, but that their approach was underpowered when working with short conserved sequences. This suggests PhyloAcc-C might be used in a complementary manner to existing methodologies, potentially extracting further insight from a given sequencing data set. Bearing this in mind, it is interesting that our analysis appeared to highlight alternative biological themes that are not present in the results of the above studies, but that do seem plausibly related to longevity and body size. These themes are a prevalence of associations with skeletal genes and genes relating to exploratory process.

When examining our top 25 LLL loci we noticed several genes related to bone strength or bone development including Fibrillin 2 (FBN2). We note that FBN2 is specifically associated with congenital contractural arachnodactyly, i.e., a particularly tall long-limbed phenotype, with long slender fingers and toes. Such non-lethal but body size-related phenotypic differences do seem to be the kind of effects that one would *a priori* imagine to be associated with true LLL loci. In the case of exploratory processes, we note that our GO analysis identified the processes ‘blood vessel endothelial cell proliferation involved in sprouting angiogenesis’ and ‘positive regulation of branching involved in lung morphogenesis’. These sort of developmental processes are exactly those thought to enhance evolvability [45, 46]. The basic reasoning is that whereas core functions are conserved across metazoa, the evolutionary flexibility of anatomical traits, such as limb shape or size, is derived from the fact that many of their constituent components are decoupled from a few fixed genetically coded features. For example, the limb is a co-ordinated collection of bone, muscle, nerves, and vasculature, but the genetic orchestration of limb development is largely achieved through cartilaginous condensations, which then select feasible arrangements of the aforementioned components. However, what works for the body during development can work against it in the case of cancer, and the importance of blood supply to tumour growth and metastasis means that as of 2018 at least 14 endothelial angiogenesis inhibitors were being used to treat cancer in the USA [47].

In conclusion, we have introduced a method that can be used to study the co-evolution of continuous traits and non-coding DNA. The method is available as an R package and users are free to modify it as they wish under the GPL. Applying the method highlighted interesting candidate LLL loci, including those related to exploratory processes, skeletal development, as well as more ‘typical’ lifespan related themes that have also been identified in other recent bioinformatics studies.

We have given some thought to future work. Longevity and size are clearly complex traits that are correlated with each other, and also with other traits, e.g., sociality [48]. Moreover, it is not unreasonable to think that thousands of enhancers and (at least) hundreds of genes are systematically involved in the evolution of these correlated related traits. PhyloAcc-C currently cannot tease apart the relative contribution of different loci to different traits of interest. One future direction would be to focus on methods for finding clusters of loci that collectively, but not always simultaneously, contribute to the variation of a trait. Another area for future work is the incorporation of more flexible null models. One way this should be attempted is by using a more realistic method to assign nucleotide rate multipliers to branches, such as relaxed, correlated, or random local clocks, or their more recent derivatives [49]. A second improvement would be to make use of alternative models of trait evolution such as those used by Uyeda and Harmon [50]. A combination of more flexible rate assignment and alternative models of trait evolution would lead to a more plausible null model overall, giving a greater confidence that a high BF indicates an interesting locus.

We see PhyloAcc-C and the other PhyloG2P methods we have discussed as first steps towards powerful tools to advance the PhyloG2P programme. Such methods will ultimately increase both our understanding of natural history and also allow us to use data from diverse species to shine a spotlight on parts of our own genome that are important for biodiversity and human health. Smith et al. [1] put it well: ‘Phylogenetics is the new genetics’.

## Supporting information

S1 Text Additional text and figures in PDF format. (PDF)

S1 Data Tables in Excel spreadsheet format. (XLSX)

S2 Data Output of GREAT analysis on LLL candidate loci in PDF format. (PDF)

## References

[1] Smith SD, Pennell MW, Dunn CW, Edwards SV. Phylogenetics is the new genetics (for most of biodiversity). Trends in Ecology & Evolution. 2020;35(5):415–425. doi: 10.1016/j.tree.2020.01.005 32294423

[2] Hiller M, Schaar BT, Indjeian VB, Kingsley DM, Hagey LR, Bejerano G. A “forward genomics” approach links genotype to phenotype using independent phenotypic losses among related species. Cell Reports. 2012;2(4):817–823. doi: 10.1016/j.celrep.2012.08.032 23022484 PMC3572205

[3] Marcovitz A, Jia R, Bejerano G. “Reverse genomics” predicts function of human conserved noncoding elements. Molecular Biology and Evolution. 2016;33(5):1358–1369. doi: 10.1093/molbev/msw001 26744417 PMC4909134

[4] Prudent X, Parra G, Schwede P, Roscito JG, Hiller M. Controlling for phylogenetic relatedness and evolutionary rates improves the discovery of associations between species’ phenotypic and genomic differences. Molecular Biology and Evolution. 2016;33(8):2135–2150. doi: 10.1093/molbev/msw098 27222536 PMC4948712

[5] Langer BE, Roscito JG, Hiller M. REforge associates transcription factor binding site divergence in regulatory elements with phenotypic differences between species. Molecular Biology and Evolution. 2018;35(12):3027–3040. doi: 10.1093/molbev/msy187 30256993 PMC6278867

[6] Partha R, Kowalczyk A, Clark NL, Chikina M. Robust method for detecting convergent shifts in evolutionary rates. Molecular Biology and Evolution. 2019;36(8):1817–1830. doi: 10.1093/molbev/msz107 31077321 PMC6657723

[7] Treaster S, Daane JM, Harris MP. Refining convergent rate analysis with topology in mammalian longevity and marine transitions. Molecular Biology and Evolution. 2021;38(11):5190–5203. doi: 10.1093/molbev/msab226 34324001 PMC8557430

[8] Hardison RC. Conserved noncoding sequences are reliable guides to regulatory elements. Trends in Genetics. 2000;16(9):369–372. doi: 10.1016/S0168-9525(00)02081-3 10973062

[9] Siepel A, Pollard KS, Haussler D. New methods for detecting lineage-specific selection. In: Annual International Conference on Research in Computational Molecular Biology 2006. Berlin: Springer Berlin Heidelberg; 2006 pp. 190–205.

[10] Rosenbloom KR, Armstrong J, Barber GP, Casper J, Clawson H, Diekhans M, et al. The UCSC genome browser database: 2015 update. Nucleic Acids Research. 2015;43(D1):D670–D681. doi: 10.1093/nar/gku1177 25428374 PMC4383971

[11] Booker BM, Friedrich T, Mason MK, VanderMeer JE, Zhao J, Eckalbar WL, et al. Bat accelerated regions identify a bat forelimb specific enhancer in the HoxD locus. PLoS Genetics. 2016;12(3):e1005738. doi: 10.1371/journal.pgen.1005738 27019019 PMC4809552

[12] Hu Z, Sackton TB, Edwards SV, Liu JS. Bayesian detection of convergent rate changes of conserved noncoding elements on phylogenetic trees. Molecular Biology and Evolution. 2019;36(5):1086–1100. doi: 10.1093/molbev/msz049 30851112 PMC6501877

[13] Lartillot N, Poujol R. A phylogenetic model for investigating correlated evolution of substitution rates and continuous phenotypic characters. Molecular Biology and Evolution. 2011;28(1):729–744. doi: 10.1093/molbev/msq244 20926596

[14] Yusuf L, Heatley MC, Palmer JP, Barton HJ, Cooney CR, Gossmann TI. Noncoding regions underpin avian bill shape diversification at macroevolutionary scales. Genome Research. 2020;30(4):553–565. doi: 10.1101/gr.255752.119 32269134 PMC7197477

[15] Kowalczyk A, Partha R, Clark NL, Chikina M. Pan-mammalian analysis of molecular constraints underlying extended lifespan. Elife. 2020;9:e51089. doi: 10.7554/eLife.51089 32043462 PMC7012612

[16] Pollard KS, Hubisz MJ, Rosenbloom KR, Siepel A. Detection of nonneutral substitution rates on mammalian phylogenies. Genome Research. 2010;20(1):110–121. doi: 10.1101/gr.097857.109 19858363 PMC2798823

[17] Cox DR, Miller HD. The theory of stochastic processes. vol. 134. CRC press; 1977.

[18] Liu JS. The collapsed Gibbs sampler in Bayesian computations with applications to a gene regulation problem. Journal of the American Statistical Association. 1994;89(427):958–966. doi: 10.1080/01621459.1994.10476829

[19] Liu J. Monte Carlo strategies in scientific computing. New York: Springer-Verlag; 2001.

[20] Felsenstein J. Evolutionary trees from DNA sequences: a maximum likelihood approach. Journal of Molecular Evolution. 1981;17:368–376. doi: 10.1007/BF01734359 7288891

[21] Kass RE, Raftery AE. Bayes factors. Journal of the American Statistical Association. 1995;90(430):773–795. doi: 10.1080/01621459.1995.10476572

[22] Wagenmakers EJ, Lodewyckx T, Kuriyal H, Grasman R. Bayesian hypothesis testing for psychologists: A tutorial on the Savage–Dickey method. Cognitive Psychology. 2010;60(3):158–189. doi: 10.1016/j.cogpsych.2009.12.001 20064637

[23] Blanchette M, Kent WJ, Riemer C, Elnitski L, Smit AF, Roskin KM, et al. Aligning multiple genomic sequences with the threaded blockset aligner. Genome Research. 2004;14(4):708–715. doi: 10.1101/gr.1933104 15060014 PMC383317

[24] Murphy WJ, Pevzner PA, O’Brien SJ. Mammalian phylogenomics comes of age. Trends in Genetics. 2004;20(12):631–639. doi: 10.1016/j.tig.2004.09.005 15522459

[25] R Core Team. R: A Language and Environment for Statistical Computing; 2021. Available from: https://www.R-project.org/.

[26] Eddelbuettel D, François R. Rcpp: Seamless R and C++ integration. Journal of Statistical Software. 2011;40:1–18. doi: 10.18637/jss.v040.i08

[27] Eddelbuettel D, Sanderson C. RcppArmadillo: Accelerating R with high-performance C++ linear algebra. Computational Statistics & Data Analysis. 2014;71:1054–1063. doi: 10.1016/j.csda.2013.02.005

[28] Paradis E, Claude J, Strimmer K. APE: analyses of phylogenetics and evolution in R language. Bioinformatics. 2004;20(2):289–290. doi: 10.1093/bioinformatics/btg412 14734327

[29] Gelman A, Rubin DB. Inference from iterative simulation using multiple sequences. Statistical Science. 1992;7(4):457–472. doi: 10.1214/ss/1177011136

[30] McLean CY, Bristor D, Hiller M, Clarke SL, Schaar BT, Lowe CB, et al. GREAT improves functional interpretation of cis-regulatory regions. Nature Biotechnology. 2010;28(5):495–501. doi: 10.1038/nbt.1630 20436461 PMC4840234

[31] Flicek P, Amode MR, Barrell D, Beal K, Brent S, Carvalho-Silva D, et al. Ensembl 2012. Nucleic Acids Research. 2012;40(D1):D84–D90. doi: 10.1093/nar/gkr991 22086963 PMC3245178

[32] Stelzer G, Rosen N, Plaschkes I, Zimmerman S, Twik M, Fishilevich S, et al. The GeneCards suite: from gene data mining to disease genome sequence analyses. Current Protocols in Bioinformatics. 2016;54(1):1–30. doi: 10.1002/cpbi.5 27322403

[33] Sollis E, Mosaku A, Abid A, Buniello A, Cerezo M, Gil L, et al. The NHGRI-EBI GWAS Catalog: knowledgebase and deposition resource. Nucleic Acids Research. 2023;51(D1):D977–D985. doi: 10.1093/nar/gkac1010 36350656 PMC9825413

[34] Thorne JL, Kishino H, Painter IS. Estimating the rate of evolution of the rate of molecular evolution. Molecular Biology and Evolution. 1998;15(12):1647–1657. doi: 10.1093/oxfordjournals.molbev.a025892 9866200

[35] Drummond AJ, Ho SYW, Phillips MJ, Rambaut A. Relaxed phylogenetics and dating with confidence. PLoS Biology. 2006;4(5):e88. doi: 10.1371/journal.pbio.0040088 16683862 PMC1395354

[36] Drummond AJ, Suchard MA. Bayesian random local clocks, or one rate to rule them all. BMC Biology. 2010;8(114):1–12. doi: 10.1186/1741-7007-8-114 20807414 PMC2949620

[37] Dorman JB, Albinder B, Shroyer T, Kenyon C. The age-1 and daf-2 genes function in a common pathway to control the lifespan of Caenorhabditis elegans. Genetics. 1995;141(4):1399–1406. doi: 10.1093/genetics/141.4.1399 8601482 PMC1206875

[38] Deelen J, Evans DS, Arking DE, Tesi N, Nygaard M, Liu X, et al. A meta-analysis of genome-wide association studies identifies multiple longevity genes. Nature Communications. 2019;10(1):3669. doi: 10.1038/s41467-019-11558-2 31413261 PMC6694136

[39] Maklakov AA, Immler S. The expensive germline and the evolution of ageing. Current Biology. 2016;26(13):R577–R586. doi: 10.1016/j.cub.2016.04.012 27404253

[40] Muntané G, Farré X, Rodríguez JA, Pegueroles C, Hughes DA, de Magalhaes JP, et al. Biological processes modulating longevity across primates: a phylogenetic genome-phenome analysis. Molecular Biology and Evolution. 2018;35(8):1990–2004. doi: 10.1093/molbev/msy105 29788292 PMC6063263

[41] Tollis M, Boddy AM, Maley CC. Peto’s Paradox: how has evolution solved the problem of cancer prevention? BMC Biology. 2017;15(60):1–5. doi: 10.1186/s12915-017-0401-7 28705195 PMC5513346

[42] Tejada-Martinez D, Avelar RA, Lopes I, Zhang B, Novoa G, De Magalhães JP, et al. Positive selection and enhancer evolution shaped lifespan and body mass in great apes. Molecular Biology and Evolution. 2022;39(2):msab369. doi: 10.1093/molbev/msab369 34971383 PMC8837823

[43] Treaster S, Deelen J, Daane JM, Murabito J, Karasik D, Harris MP. Convergent genomics of longevity in rockfishes highlights the genetics of human life span variation. Science Advances. 2023;9(2):eadd2743. doi: 10.1126/sciadv.add2743 36630509 PMC9833670

[44] Kolora SRR, Owens GL, Vazquez JM, Stubbs A, Chatla K, Jainese C, et al. Origins and evolution of extreme life span in Pacific Ocean rockfishes. Science. 2021;374(6569):842–847. doi: 10.1126/science.abg5332 34762458 PMC8923369

[45] Kirschner M, Gerhart J. Evolvability. Proceedings of the National Academy of Sciences. 1998;95(15):8420–8427. doi: 10.1073/pnas.95.15.8420 9671692 PMC33871

[46] Kirschner MW, Gerhart JC. The plausibility of life: Resolving Darwin’s dilemma. Yale University Press; 2005.

[47] NIH National Cancer Institute. Angiogenesis Inhibitors; 2018. https://www.cancer.gov/about-cancer/treatment/types/immunotherapy/angiogenesis-inhibitors-fact-sheet.

[48] Zhu P, Liu W, Zhang X, Li M, Liu G, Yu Y, et al. Correlated evolution of social organization and lifespan in mammals. Nature Communications. 2023;14(1):372. doi: 10.1038/s41467-023-35869-7 36720880 PMC9889386

[49] Fisher AA, Ji X, Nishimura A, Lemey P, Baele G, Suchard MA. Shrinkage-based random local clocks with scalable inference. Molecular Biology and Evolution. 2023;40(11). doi: 10.1093/molbev/msad242 37950885 PMC10665039

[50] Uyeda JC, Harmon LJ. A novel Bayesian method for inferring and interpreting the dynamics of adaptive landscapes from phylogenetic comparative data. Systematic Biology. 2014;63(6):902–918. doi: 10.1093/sysbio/syu057 25077513

