# Supplementary material for: A phylogenetic method linking nucleotide substitution rates to rates of continuous trait evolution
Source: PLoS Comput Biol. 2024 Apr 24;20(4):e1011995. doi: 10.1371/journal.pcbi.1011995 (PMC11078400; doi:10.1371/journal.pcbi.1011995)
Supplement: S2 Data — (PDF) [file pcbi.1011995.s003.pdf]

Ontology	# Term Name	Term ID	Hyper Rank	Hyper Raw P-Value	Hyper Bonferroni P-Value	Hyper FDR Q-Val	Hyper Fold Enrichment	Hyper Expected	Hyper Foreground Region Hits	Hyper Total Regions	Hyper Region Set Coverage	Hyper Term Region Coverage	Hyper Foreground Gene Hits	Hyper Background Gene Hits	Total Genes Annotated
Ensembl Genes	No results meet your chosen criteria.														
GO Biological Process	positive regulation of cardiocyte differentiation	GO:1905209	1	1.28778e-9	1.69459e-5	1.69459e-5	3.2619	11.0366	36	1362	3.25%	2.64%	9	22	25
	positive regulation of cardiac muscle tissue development	GO:0055025	2	1.77092e-8	2.33035e-4	1.16518e-4	2.5377	18.1269	46	2237	4.15%	2.06%	13	36	40
	positive regulation of striated muscle tissue development	GO:0045844	3	2.39969e-8	3.15775e-4	1.05258e-4	2.3309	22.7377	53	2806	4.78%	1.89%	16	56	64
	positive regulation of cardiac muscle cell differentiation	GO:2000727	4	4.85776e-8	6.39232e-4	1.59808e-4	3.2683	8.8730	29	1095	2.61%	2.65%	7	14	17
	blood vessel endothelial cell proliferation involved in sprouting angiogenesis	GO:0002043	6	2.06984e-7	2.72370e-3	4.53949e-4	5.3811	2.7875	15	344	1.35%	4.36%	2	7	7
	positive regulation of branching involved in lung morphogenesis	GO:0061047	7	2.60776e-7	3.43155e-3	4.90221e-4	5.7020	2.4553	14	303	1.26%	4.62%	3	4	4
	regulation of cardiocyte differentiation	GO:1905207	9	4.02047e-7	5.29054e-3	5.87838e-4	2.4082	17.0249	41	2101	3.70%	1.95%	13	36	41
	positive regulation of muscle tissue development	GO:1901863	11	9.21456e-7	1.21254e-2	1.10231e-3	2.0692	25.6143	53	3161	4.78%	1.68%	16	57	65
	regulation of cell proliferation involved in outflow tract morphogenesis	GO:1901963	12	1.18731e-6	1.56238e-2	1.30198e-3	5.9474	2.0177	12	249	1.08%	4.82%	2	2	2
	negative regulation of	GO:2000242	14	3.08082e-6	4.05406e-2	2.89575e-3	2.7744	9.7320	27	1201	2.43%	2.25%	8	31	54

	reproductive process														
	regulation of cardiac muscle cell differentiation	GO:2000725	15	3.99040e-6	5.25097e-2	3.50064e-3	2.4415	13.5162	33	1668	2.98%	1.98%	10	24	29
	smooth muscle cell differentiation	GO:0051145	16	4.41017e-6	5.80334e-2	3.62709e-3	2.3922	14.2131	34	1754	3.07%	1.94%	12	30	32
	tendon development	GO:0035989	17	5.50877e-6	7.24899e-2	4.26411e-3	5.6327	1.9529	11	241	0.99%	4.56%	2	4	5
	regulation of alkaline phosphatase activity	GO:0010692	18	5.64689e-6	7.43074e-2	4.12819e-3	3.7001	4.5945	17	567	1.53%	3.00%	4	7	9
	astrocyte development	GO:0014002	19	6.79312e-6	8.93907e-2	4.70477e-3	3.4872	5.1618	18	637	1.62%	2.83%	4	20	27
	organ induction	GO:0001759	20	7.32160e-6	9.63449e-2	4.81725e-3	2.9171	7.8844	23	973	2.07%	2.36%	6	15	15
	positive regulation of cardiac muscle tissue growth	GO:0055023	21	8.15244e-6	1.07278e-1	5.10847e-3	2.3948	13.3622	32	1649	2.89%	1.94%	8	27	30
	cardiac muscle cell differentiation	GO:0055007	22	9.00607e-6	1.18511e-1	5.38686e-3	2.0357	22.1056	45	2728	4.06%	1.65%	18	64	77
	endocrine pancreas development	GO:0031018	23	9.13124e-6	1.20158e-1	5.22426e-3	2.2176	16.6846	37	2059	3.34%	1.80%	12	32	38
	trachea formation	GO:0060440	24	9.13554e-6	1.20215e-1	5.00894e-3	4.8714	2.4634	12	304	1.08%	3.95%	2	6	6
GO Cellular Component	BBSome	GO:0034464	1	9.29158e-6	1.60558e-2	1.60558e-2	5.9331	1.6855	10	208	0.90%	4.81%	3	9	10
	ciliary transition zone	GO:0035869	2	9.85734e-6	1.70335e-2	8.51674e-3	3.5438	4.7971	17	592	1.53%	2.87%	7	37	56
GO Molecular Function	No results meet your chosen criteria.														
Human Phenotype	Abdominal distention	HP:0003270	1	5.85501e-7	3.90705e-3	3.90705e-3	3.6501	5.7533	21	710	1.89%	2.96%	4	21	34
	Polymicrogyria	HP:0002126	2	1.38566e-6	9.24650e-3	4.62325e-3	3.0486	8.2005	25	1012	2.25%	2.47%	10	39	67
	Uplifted earlobe	HP:0009909	3	9.47504e-6	6.32270e-2	2.10757e-2	3.4017	5.2914	18	653	1.62%	2.76%	2	3	4
	Protuberant abdomen	HP:0001538	4	1.18974e-5	7.93916e-2	1.98479e-2	5.7667	1.7341	10	214	0.90%	4.67%	2	12	17
	Pulmonic stenosis	HP:0001642	5	1.53953e-5	1.02733e-1	2.05465e-2	2.4379	11.8955	29	1468	2.61%	1.98%	11	38	55
	Abnormality of the	HP:0002518	6	1.58370e-5	1.05681e-1	1.76134e-2	3.7701	3.9787	15	491	1.35%	3.05%	4	17	29

	periventricular white matter														
	Excessive salivation	HP:0003781	8	2.49276e-5	1.66342e-1	2.07927e-2	2.7704	7.9412	22	980	1.98%	2.24%	6	18	33
	Abnormal pulmonary valve morphology	HP:0001641	9	4.27651e-5	2.85372e-1	3.17080e-2	2.2644	13.2488	30	1635	2.71%	1.83%	12	43	63
	Severe muscular hypotonia	HP:0006829	10	4.43036e-5	2.95638e-1	2.95638e-2	3.8658	3.3628	13	415	1.17%	3.13%	5	27	39
	Abnormality of the fingernails	HP:0001231	14	6.28269e-5	4.19244e-1	2.99460e-2	3.5187	3.9787	14	491	1.26%	2.85%	5	8	13
	Drooling	HP:0002307	16	7.05757e-5	4.70951e-1	2.94345e-2	2.7272	7.3334	20	905	1.80%	2.21%	5	15	29
	Cone-shaped epiphysis	HP:0010579	17	7.40366e-5	4.94046e-1	2.90615e-2	2.5710	8.5570	22	1056	1.98%	2.08%	6	21	29
	Absent in utero rib ossification	HP:0006615	18	8.24000e-5	5.49855e-1	3.05475e-2	6.8560	1.0210	7	126	0.63%	5.56%	1	1	1
	Absent in utero ossification of vertebral bodies	HP:0008435	18	8.24000e-5	5.49855e-1	3.05475e-2	6.8560	1.0210	7	126	0.63%	5.56%	1	1	1
	Increased nuchal translucency	HP:0010880	18	8.24000e-5	5.49855e-1	3.05475e-2	6.8560	1.0210	7	126	0.63%	5.56%	1	1	2
	Unossified sacrum	HP:0030290	18	8.24000e-5	5.49855e-1	3.05475e-2	6.8560	1.0210	7	126	0.63%	5.56%	1	1	1
	Abnormal liver lobulation	HP:0100752	18	8.24000e-5	5.49855e-1	3.05475e-2	6.8560	1.0210	7	126	0.63%	5.56%	1	1	1
	Broad forehead	HP:0000337	23	8.38995e-5	5.59861e-1	2.43418e-2	2.0148	17.8676	36	2205	3.25%	1.63%	8	35	49
	Rod-cone dystrophy	HP:0000510	24	8.68398e-5	5.79482e-1	2.41451e-2	2.1432	14.4643	31	1785	2.80%	1.74%	12	79	127
	Abnormal rib ossification	HP:0012306	26	1.05185e-4	7.01897e-1	2.69960e-2	6.5943	1.0615	7	131	0.63%	5.34%	1	2	5
Mouse Phenotype Single KO	ostium primum atrial septal defect	MP:0010404	1	2.02072e-6	1.85037e-2	1.85037e-2	2.9837	8.3787	25	1034	2.25%	2.42%	7	16	17
	abnormal Meibomian gland morphology	MP:0005252	2	4.47180e-6	4.09483e-2	2.04742e-2	4.1975	3.5735	15	441	1.35%	3.40%	3	12	16
	abnormal skin sebaceous gland morphology	MP:0009535	3	5.54204e-6	5.07484e-2	1.69161e-2	4.1227	3.6383	15	449	1.35%	3.34%	3	13	18
	ectopic ureter	MP:0011486	4	9.31434e-6	8.52914e-2	2.13228e-2	4.1935	3.3385	14	412	1.26%	3.40%	2	3	3
	abnormal pericardial cavity morphology	MP:0012501	6	1.32280e-5	1.21129e-1	2.01881e-2	2.0617	20.3715	42	2514	3.79%	1.67%	22	91	118

	pericardial edema	MP:0001787	7	1.45641e-5	1.33364e-1	1.90520e-2	2.2571	15.0639	34	1859	3.07%	1.83%	16	58	76
	abnormal interatrial septum morphology	MP:0000282	8	1.61802e-5	1.48162e-1	1.85203e-2	2.0863	19.1722	40	2366	3.61%	1.69%	15	77	96
	atrial septal defect	MP:0010403	10	1.83702e-5	1.68216e-1	1.68216e-2	2.1210	17.9162	38	2211	3.43%	1.72%	14	66	82
	decreased cornea thickness	MP:0005543	11	3.43532e-5	3.14572e-1	2.85975e-2	3.7235	3.7599	14	464	1.26%	3.02%	4	15	17
	abnormal prostate gland anterior lobe morphology	MP:0001163	13	4.43036e-5	4.05689e-1	3.12068e-2	3.8658	3.3628	13	415	1.17%	3.13%	3	13	14
	abnormal brain ependyma motile cilium morphology	MP:0011056	14	5.23538e-5	4.79404e-1	3.42432e-2	5.4179	1.6612	9	205	0.81%	4.39%	4	10	16
	abnormal ependyma motile cilium morphology	MP:0011059	14	5.23538e-5	4.79404e-1	3.42432e-2	5.4179	1.6612	9	205	0.81%	4.39%	4	10	18
	absent Meibomian glands	MP:0006236	16	5.40483e-5	4.94921e-1	3.09325e-2	9.1413	0.6564	6	81	0.54%	7.41%	2	5	8
	atrioventricular septal defect	MP:0010412	17	5.91320e-5	5.41472e-1	3.18513e-2	2.1898	14.1563	31	1747	2.80%	1.77%	9	55	75
	abnormal head mesenchyme morphology	MP:0011260	18	6.18881e-5	5.66709e-1	3.14839e-2	3.1899	5.0159	16	619	1.44%	2.58%	5	22	28
	absent subcutaneous adipose tissue	MP:0008843	19	9.01103e-5	8.25140e-1	4.34284e-2	3.8465	3.1197	12	385	1.08%	3.12%	3	15	19
	abnormal kidney interstitium morphology	MP:0011425	20	9.72795e-5	8.90788e-1	4.45394e-2	2.3117	11.2473	26	1388	2.34%	1.87%	11	29	49
	abnormal subcommissural organ morphology	MP:0009716	22	1.03657e-4	9.49191e-1	4.31450e-2	8.1368	0.7374	6	91	0.54%	6.59%	2	4	4
	absent tracheal cartilage rings	MP:0004553	24	1.16811e-4	1.00000	4.45681e-2	3.3161	4.2218	14	521	1.26%	2.69%	2	3	3
	abnormal atrioventricular septum morphology	MP:0010592	25	1.33537e-4	1.00000	4.89119e-2	2.0632	15.5096	32	1914	2.89%	1.67%	10	58	78
Mouse Phenotype	small interparietal bone	MP:0004384	1	9.73658e-8	9.30914e-4	9.30914e-4	3.0878	9.7158	30	1199	2.71%	2.50%	10	21	24

	complete atrioventricular septal defect	MP:0010413	2	3.34438e-7	3.19756e-3	1.59878e-3	3.6394	6.0450	22	746	1.98%	2.95%	4	20	30
	abnormal vertebral body development	MP:0005227	3	4.56862e-7	4.36806e-3	1.45602e-3	4.4637	3.8085	17	470	1.53%	3.62%	3	10	12
	pericardial edema	MP:0001787	4	2.09009e-6	1.99833e-2	4.99584e-3	2.0113	26.3517	53	3252	4.78%	1.63%	23	77	102
	abnormal ureterovesical junction morphology	MP:0011488	6	3.07914e-5	2.94396e-1	4.90661e-2	3.2375	5.2509	17	648	1.53%	2.62%	3	5	6
	abnormal intraocular pressure	MP:0005257	7	3.96052e-5	3.78665e-1	5.40950e-2	2.6160	8.7920	23	1085	2.07%	2.12%	9	23	26
	increased hepatocyte apoptosis	MP:0003887	8	4.54044e-5	4.34111e-1	5.42639e-2	2.8175	7.0984	20	876	1.80%	2.28%	8	49	69
	pancreas cysts	MP:0003336	9	4.55985e-5	4.35968e-1	4.84409e-2	3.2745	4.8862	16	603	1.44%	2.65%	5	13	21
	absent Meibomian glands	MP:0006236	10	5.40483e-5	5.16756e-1	5.16756e-2	9.1413	0.6564	6	81	0.54%	7.41%	2	5	8
	ostium primum atrial septal defect	MP:0010404	11	5.46298e-5	5.22316e-1	4.74832e-2	2.4466	10.2182	25	1261	2.25%	1.98%	7	22	23
	fused kidneys	MP:0003605	12	5.73572e-5	5.48392e-1	4.56993e-2	4.0351	2.9739	12	367	1.08%	3.27%	4	6	6
	common atrioventricular valve	MP:0010607	13	6.01714e-5	5.75299e-1	4.42538e-2	2.6800	7.8358	21	967	1.89%	2.17%	6	16	18
	abnormal canal of Schlemm morphology	MP:0005204	14	6.11573e-5	5.84725e-1	4.17661e-2	2.8456	6.6771	19	824	1.71%	2.31%	6	13	14
	macrophthalmia	MP:0001296	15	7.62318e-5	7.28852e-1	4.85902e-2	3.0056	5.6561	17	698	1.53%	2.44%	5	14	18
	small zygomatic bone	MP:0004468	16	9.34700e-5	8.93667e-1	5.58542e-2	2.9548	5.7533	17	710	1.53%	2.39%	7	9	10
	abnormal head mesenchyme morphology	MP:0011260	17	9.99293e-5	9.55424e-1	5.62014e-2	2.3533	10.6233	25	1311	2.25%	1.91%	11	37	45
	abnormal subcommissural organ morphology	MP:0009716	18	1.03657e-4	9.91068e-1	5.50594e-2	8.1368	0.7374	6	91	0.54%	6.59%	2	4	4
	exocrine pancreas atrophy	MP:0009164	19	1.14097e-4	1.00000	5.74149e-2	4.0401	2.7227	11	336	0.99%	3.27%	4	7	8
	increased cardiomyocyte apoptosis	MP:0003222	21	1.35130e-4	1.00000	6.15228e-2	2.4637	8.9298	22	1102	1.98%	2.00%	9	52	60
	small vertebral body	MP:0004670	22	1.40711e-4	1.00000	6.11516e-2	2.8543	5.9559	17	735	1.53%	2.31%	3	14	17

The test set contains 1,109 (1%) of all 136,859 regions.	The test set picked 1,072 genes, the background set picked 11,393 genes.
Ensembl Genes has 18,777 terms covering 18,777 (100%) of all 18,777 genes.	18,777 ontology terms were tested (100%) using an annotation count range of [1, 1000].
GO Biological Process has 13,159 terms covering 16,804 (89%) of all 18,777 genes.	13,159 ontology terms were tested (100%) using an annotation count range of [1, 1000].
GO Cellular Component has 1,728 terms covering 17,911 (95%) of all 18,777 genes.	1,728 ontology terms were tested (100%) using an annotation count range of [1, 1000].
GO Molecular Function has 4,222 terms covering 16,729 (89%) of all 18,777 genes.	4,222 ontology terms were tested (100%) using an annotation count range of [1, 1000].
Human Phenotype has 6,673 terms covering 3,390 (18%) of all 18,777 genes.	6,673 ontology terms were tested (100%) using an annotation count range of [1, 1000].
Mouse Phenotype Single KO has 9,157 terms covering 9,525 (51%) of all 18,777 genes.	9,157 ontology terms were tested (100%) using an annotation count range of [1, 1000].
Mouse Phenotype has 9,561 terms covering 9,709 (52%) of all 18,777 genes.	9,561 ontology terms were tested (100%) using an annotation count range of [1, 1000].

GREAT version 4.0.4
Species assembly: hg38
Association rule: Basal+extension: 5000 bp upstream, 1000 bp downstream, 1000000 bp max extension, curated regulatory domains included
